# TIGAR Attenuates High Glucose-Induced Neuronal Apoptosis *via* an Autophagy Pathway

**DOI:** 10.3389/fnmol.2019.00193

**Published:** 2019-08-13

**Authors:** Wenjuan Zhou, Yuan Yao, Jinxing Li, Dong Wu, Man Zhao, Zongting Yan, Aimei Pang, Liang Kong

**Affiliations:** ^1^Key Laboratory of the Ministry of Education for Experimental Teratology, Shandong Provincial Key Laboratory of Mental Disorders, Department of Human Anatomy and Histoembryology, School of Basic Medical Sciences, Shandong University, Jinan, China; ^2^Department of Physical Education, Shanghai Normal University, Shanghai, China; ^3^Department of Clinical Laboratory, Affiliated Hospital of Shandong University of Traditional Chinese Medicine, Jinan, China

**Keywords:** hyperglycemia, TIGAR, neuronal apoptosis, autophagy, NOS1

## Abstract

Hyperglycemia-induced neuronal apoptosis is one of the important reasons for diabetic neuropathy. Long-time exposure to high glucose accelerates many aberrant glucose metabolic pathways and eventually leads to neuronal injury. However, the underlying mechanisms of metabolic alterations remain unknown. TP53-inducible glycolysis and apoptosis regulator (TIGAR) is an endogenous inhibitor of glycolysis and increases the flux of pentose phosphate pathway (PPP) by regulating glucose 6-phosphate dehydrogenase (G6PD). TIGAR is highly expressed in neurons, but its role in hyperglycemia-induced neuronal injury is still unclear. In this study, we observed that TIGAR and G6PD are decreased in the hippocampus of streptozotocin (STZ)-induced diabetic mice. Correspondingly, in cultured primary neurons and Neuro-2a cell line, stimulation with high glucose induced significant neuronal apoptosis and down-regulation of TIGAR expression. Overexpression of TIGAR reduced the number of TUNEL-positive neurons and prevented the activation of Caspase-3 in cultured neurons. Furthermore, enhancing the expression of TIGAR rescued high glucose-induced autophagy impairment and the decrease of G6PD. Nitric oxide synthase 1 (NOS1), a negative regulator of autophagy, is also inhibited by overexpression of TIGAR. Inhibition of autophagy abolished the protective effect of TIGAR in neuronal apoptosis in Neuro-2a. Importantly, overexpression of TIGAR in the hippocampus ameliorated STZ-induced cognitive impairment in mice. Therefore, our data demonstrated that TIGAR may have an anti-apoptosis effect *via* up-regulation of autophagy in diabetic neuropathy.

## Introduction

Diabetes mellitus (DM) is a systemic metabolic, disease and more than 60% patients are subjected to diabetic neuropathy, leading to severe neuronal injury and cognitive impairments (Vincent and Feldman, 2004; Sullivan et al., 2007; Vincent et al., 2011). Chronic hyperglycemia is the most prevalent characteristic of diabetes patients. Although the brain is a high energy-demanding organ, consuming excessive amounts of glucose leads to an overloaded process. Many aberrant glucose metabolic pathways may be activated or up-regulated under chronic hyperglycemia (Luo et al., 2016). However, the underlying mechanisms of metabolic alterations and glucotoxicity in diabetic neuropathy still remain unclear. In fact, under hyperglycemic conditions, the dormant polyol pathway is activated and causes reductive stress by generation of excess NADH (Yagihashi et al., 2001). Overload of the electron transport chain and oxidative phosphorylation also lead to mitochondrial dysfunction and oxidative stress (Edwards et al., 2008; Yan, 2014). In contrast, the activity of glucose 6-phosphate dehydrogenase (G6PD), a rate-limiting enzyme in the pentose phosphate pathway (PPP), is decreased after stimulation with high glucose (Okouchi et al., 2005; Sun et al., 2019). PPP has been regarded as a main metabolic pathway to regenerate glutathione at the expense of NADPH and protect neurons from oxidative stress (Herrero-Mendez et al., 2009). Therefore, understanding how to transform aberrant glucose metabolic pathways into antioxidant metabolic pathways may provide new therapeutic strategies for the treatment of diabetic neuropathy.

TP53-inducible glycolysis and apoptosis regulator (TIGAR) is an important bisphosphatase and widely distributed in neurons (Li et al., 2014). TIGAR decreases glycolytic flux by reducing the levels of Fru-2, 6-P_2_ and increases PPP by up-regulation of G6PD (Bensaad et al., 2006; Li et al., 2014). Emerging evidence has proved that TIGAR plays crucial roles in both physiological and pathological processes (Li et al., 2014; Zhou et al., 2019). For instance, TIGAR is a key regulator during embryonic brain development. It can reprogram the glucose metabolism pathway from glycolysis to mitochondrial oxidative phosphorylation and promote neural stem cell differentiation through acetyl-CoA-mediated histone acetylation (Zhou et al., 2019). After ischemia/reperfusion, TIGAR was rapidly up-regulated in the ischemic cortex of mice (Li et al., 2014). Overexpression of TIGAR protects ischemic brain damage *via* enhancing PPP flux and rescuing dysfunction of mitochondria. However, the effect of TIGAR in hyperglycemia-induced neuronal apoptosis remains elusive. Whether TIGAR could reduce glucotoxicity by alteration of metabolic pathways is unclear.

Autophagy, a conserved self-digestion pathway, scavenges protein aggregates and damaged organelles in physiological processes. Autophagy dysfunction has been found in many neurodegenerative disorders (Nixon, 2013). In diabetes, autophagic activity can be destroyed by chronic hyperglycemia (Li et al., 2017; Yerra and Kumar, 2017). Studies have reported that autophagy dysfunction is one significant cause of hyperglycemia-induced neuronal apoptosis and cognitive impairments (Xue et al., 2016; Li et al., 2017; Yerra and Kumar, 2017). However, how neuronal autophagy is regulated under chronic hyperglycemic condition remains largely unknown. It has been shown that different glucose metabolic pathways and metabolic regulators can direct autophagic activity in multiple types of cells (Desideri et al., 2014; Strohecker et al., 2015; Tan and Miyamoto, 2015). TIGAR has been proved to be an important regulator of autophagy (Hoshino et al., 2012; Xie et al., 2014). In cancer cells, TIGAR suppressed reactive oxygen species (ROS) levels and inhibited autophagy in response to metabolic stress (Bensaad et al., 2009). In an ischemia and reperfusion stroke model, TIGAR inhibited activation of autophagy through the mTOR-S6KP70 signaling pathway and further prevented ischemia and reperfusion-induced neuronal injury (Zhang et al., 2019). However, under hyperglycemic conditions, the effect of TIGAR on diabetes-induced neuronal autophagy impairment is still unclear.

In this study, we aimed to investigate the effect of TIGAR in hyperglycemia-induced neuronal apoptosis and autophagy impairment. Fully understanding the mechanism of TIGAR in high glucose-induced neuronal injury may offer a new therapeutic approach in diabetic neuropathy.

## Materials and Methods

### Animals and Treatment

Adult male C57BL/6 mice weighing 25–30 g were used in this study. Mice were raised under standard house circumstances (22 ± 1°C and enough food and water) with a 12:12 light/dark cycle. All procedures conformed to the National Institutes of Health *Guide for the Care and Use of Laboratory Animals* and were allowed by the Institutional Animal Care and Use Committees of Shandong University.

Mice were randomly divided into two groups: a control group and the diabetes group. After fasting 12 h, mice in two groups were administered by a single intraperitoneal injection of vehicle or streptozotocin (STZ, Sigma) solution, respectively. According to a previous study, STZ was dissolved in 0.1 M sodium citrate buffer (pH 4.4) and a dose of 220 mg/kg was used for injection (Ferber et al., 2000; Shi et al., 2018). Fasting blood glucose levels were examined 3 days after injection of STZ using an FAD-GDH System (Sanocare, Changsha, China). Mice in the diabetes group with blood glucose ≥16.7 mM were used in the following experiment. After 4 weeks post-STZ injection, mice were sacrificed and brains were obtained for western blot and immunohistochemistry.

### Cell Cultures

According to a previous study, primary hippocampal neurons were prepared from E18 mice. Briefly, mouse hippocampus was isolated and digested with 0.05% trysin. Neurons were cultured with neurobasal medium (Gibco) containing 2% B27, 1% penicillin/streptomycin solution and 0.5 mM L-glutamine. Neurons were plated on poly-D-lysine-coated cell dishes and cultured for 7 days. Mouse Neuro-2a neuroblastoma cells were cultured in MEM medium supplemented with 10% fetal bovine serum, 2 mM L-glutamine and 1% penicillin/streptomycin solution. Cells were maintained at 37°C under 5% CO_2_ atmosphere. High glucose culture medium with 100 mM glucose was used to stimulate primary hippocampal neurons for 72 h. A total of 50 mM glucose was used to stimulate Neuro-2a cells for 48 h. Chloroquine diphosphate (CQ; MedChemExpress, 10 μM) and 3-MA (Sigma, 2 mM) were used as an inhibitor of autophagy in this study.

### Immunofluorescence

At the time of sacrifice, mice were anesthetized with an overdose of barbiturate and perfused with 4% paraformaldehyde. Mouse brains were removed and infiltrated with 30% sucrose. Serial coronal sections of 30 μM were cut using a freezing microtome. Cultured cells were fixed with 4% paraformaldehyde for 30 min. Cells and brain slices were blocked and permeabilized in 5% goat serum solution containing 0.3% Triton X-100 for 2 h at room temperature. Then, slices were incubated with primary antibodies against rabbit anti-TIGAR (1:500, Abcam) and rabbit anti-cleaved Caspase-3 (1:500, CST) overnight at 4°C. The cells and sections were washed in PBS three times and incubated with secondary antibodies conjugated to Alexa Fluor 488 or Alexa Fluor 594 for 1 h at room temperature. Before fluorescent photograph, the cells and brain sections were stained with 2 μg/ml 4′,6-diamidino-2-phenylindole (DAPI). Photos were captured with a fluorescence microscope (Olympus IX71). The FITC green and TRITC Red Fluorescein *in situ* Apoptosis Detection Kit (KeyGEN BioTECH, China) was used for TUNEL staining. TUNEL positive cells were counted under the fluorescent microscope.

### RNA Interference Plasmid Constructs and Lentivirus Packaging

TIGAR-related plasmids were designed as previously described (Zhou et al., 2019). In brief, the shRNA sequences of TIGAR were first ligated into pSilencer 1.0 and then inserted into PGW vector with the U6 promoter. The TIGAR shRNA sequences are listed in Table 1. To enhance the expression of TIGAR, TIGAR sequences were inserted into pcDNA3.1 and pUltra. The transfer plasmids (pSilencer-siTIGAR or pUltra-TIGAR) and package plasmids (pMDL/pRRE, VSV-G, and pRSV-REV) were transfected into 293T to harvest high titer lentiviruses.

**Table 1:** Primer sequence.

Name	Forward primer	Reverse primer
*Tigar*	AGGGCAGAGAGAAAGCGT	TGCCACCTTTGGGATTC
*G6pd*	CAGCCCAATGAGGCAGTA	CCACAGAAGACATCCAGG
*Nos1*	GCGTTCGTGATTACTGTG	GTCACCTTGTCACTCTGGA
*β-actin*	CGTTGACATCCGTAAAGACCTC	CCACCGATCCACACAGAGTAC
*Tigar shRNA*	TTAGCAGCCAGCATCTTAGTTCAAGAGACTAAGATGCTGGCTG CTAATTTTTT	AATTAAAAAATTAGCAGCCAGCATCTTAGTCTCTTGAACTAAGATGCT GGCTGCTAAGGCC

### RNA Isolation and Real-Time Quantitative PCR

Cultured neurons and brain tissues were collected and homogenized in TRIZOL solution (Invitrogen). Total RNA was extracted and then its concentration was measured by a spectrophotometer. RNA was converted to cDNA by using the RevertAid™ First Strand cDNA Synthesis Kit (Thermo Fisher Scientific), and real time PCR was performed by the SYBR Green Realtime PCR Master Mix (TOYOBO). The expression of *β-actin* gene represented internal controls, and the 2^−ΔΔCT^ method was used to calculate relative expression of genes. The gene primer sequences are shown in Table 1.

### Western Blotting

In brief, cultured cells and brain tissues were homogenized in RIRA buffer supplemented with protease and phosphatase inhibitors on ice. The homogenates were centrifuged at 12,000 rpm for 20 min at 4°C. The supernatants were collected and quantified using the bicinchoninic acid (BCA) assay (Pierce Biotechnology). The samples were mixed with loading buffer and boiled for 5 min. Equal amounts of proteins were loaded to each well and separated by SDS-PAGE. Primary antibodies were used at the following dilutions: rabbit anti-TIGAR (1:500, Abcam), mouse anti-Bcl-2 (1:500, Santa), rabbit anti-Bax (1:1,000, CST), rabbit anti-LC3B (1:1,000, CST), rabbit anti-Beclin-1 (1:500, CST), rabbit anti-Caspase-3 (1:1,000, CST), rabbit anti-cleaved-Caspase-3 (1:500, CST), rabbit anti-β-actin (1:2,000, CST), rabbit anti-P62 (1:1,000, CST), rabbit anti-nitric oxide synthase 1 (NOS1; 1:100, CST) and rabbit anti-G6PD (1:1,000, CST).

### Quantification of Intracellular Nitric Oxide (NO) and NADPH

Nitric oxide (NO) quantification was conducted using an NO assay kit (Beyotime Institute of Biotechnology, Shanghai, China). Briefly, 1 × 10^6^ cells were treated with cell and tissue lysis (for NO detection). The supernatant was centrifuged to remove sediments. A total of 50 μl/well of the supernatant and standard were added into a 96-well plate. Griess Reagent I and Griess Reagent II of equal volume were added into each well. Then the absorption was measured at 540 nm.

NADPH was measured using a NADPH assay kit with WST-8 (Beyotime Institute of Biotechnology, Shanghai, China) according to the manufacturer’s protocol. Briefly, 1 × 10^6^ cells were lysed with 200 μl NADPH extracting solution. The supernatant was harvested by centrifuging for 10 min at 12,000 rpm at 4°C. Then the supernatant was heated at 60°C to decompose NADP^+^. After centrifugation, 50 μl supernatant and 100 μl G6PDH working solution were added to each well and hatched in the dark for 10 min at 37°C. Each well was then mixed with 10 μl 1-mPMS and incubated at 37°C for 15 min. At this time, an orange formazan was formed and the absorbance at 450 nm was measured.

### Stereotaxic Surgery and Microinjection

Mice were anesthetized by 5% chloral hydrate (400 mg/kg, i.p.). Then mice were placed in a stereotaxic apparatus and received dorsal hippocampus (DH) infusions. The coordinates were as follows: anteroposterior, −1.7 mm; lateral, ±1.5 mm; dorsoventral, −2.3 mm. TIGAR overexpressing lentivirus (1 μl per side) was infused into the DH.

Locomotor activity was measured in a square box (40 cm × 40 cm × 35 cm) and mice were placed in the center of the field with 10 min exploration. The total distance traveled in the arena was recorded as a measure of locomotor activity.

The novel object recognition memory (ORM) and location-dependent ORM (OLM) tests were performed as described in the protocol previously (McQuown et al., 2011; Yan et al., 2015). Briefly, on the first day, mice were habituated in the experimental environment for 10 min. On the second day, mice were exposed to two identical objects for 5 min for familiarization. On the third day (retention test), mice were exposed to objects for 5 min. For ORM, one familiar object A was changed into a new object B in the same location. For OLM, mice were exposed to two familiar objects, but one of the familiar objects was placed in the new location. All training and testing trials were recorded and analyzed by an observer blind to the treatment condition. The exploration time was recorded when the mouse was directed at the object within 1 cm or when its nose was in contact with the object. The discrimination index was calculated by T_novel_/(T_novel_ + T_familiar_).

### Statistical Analysis

Statistical analysis was performed with SPSS 19.0 program. The results are presented as the mean ± SEM from at least three independent experiments. Data difference comparisons were analyzed by Student’s *t*-test, one-way or two-way ANOVA followed by the LSD or Dunnett’s T3 *post hoc* test. The significance level was set at 0.05.

## Results

### TIGAR Were Decreased in the Hippocampus of STZ-Induced Diabetic Mice and High Glucose-Treated Primary Neurons

**Figure 1:**
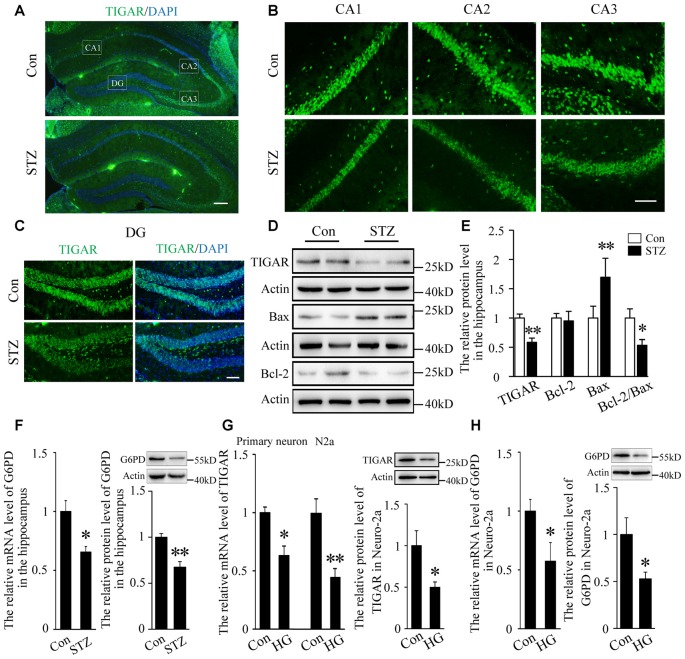
TP53-inducible glycolysis and apoptosis regulator (TIGAR) expression in the hippocampus of streptozotocin (STZ)-induced diabetic mice. **(A)** Immunofluorescent staining of TIGAR expression in the hippocampus in control group and STZ-treated group. Scale bar = 200 μm. **(B,C)** The expression of TIGAR in the CA1, CA2, CA3 and dentate gyrus (DG) of the hippocampus. Scale bar = 50 μm. **(D,E)** Western blot analysis of TIGAR, Bcl-2 and Bax in the hippocampus of each group (*n* = 6 per group). **(F)** Quantification of the mRNA and protein levels of glucose 6-phosphate dehydrogenase (G6PD) in each group (*n* = 4 per group). **(G)** Quantification of the mRNA and protein levels of TIGAR in high glucose-treated hippocampal primary neurons and Neuro-2a cells (*n* = 3–6 per group). **(H)** Quantification of the mRNA and protein levels of G6PD in high glucose-treated Neuro-2a cells (*n* = 3–4 per group). *p < 0.05, **p < 0.01, *t*-test. Data represent the mean of at least three independent experiments.

Studies have shown that neuronal apoptosis in the hippocampus was closely related with cognitive impairments in diabetic mice (Guo et al., 2014; Shi et al., 2018). Blood glucose was examined 3 days after injection of STZ (Supplementary Figure 1A). Four weeks after STZ injection, the expression of TIGAR in the hippocampus of diabetic mice was investigated. By immunofluorescent staining and western blotting for TIGAR, we observed that TIGAR was significantly decreased in the hippocampus of STZ-treated mice (Figures 1A–E). Pro-apoptotic Bax and anti-apoptotic Bcl-2 proteins were detected in the hippocampus. Compared with control mice, the level of Bax was increased and the ratio of Bcl-2/Bax was decreased in the hippocampus of STZ-treated mice (Figures 1D,E), suggesting that neuronal apoptosis was aggravated in hyperglycemic mice. G6PD, a rate-limiting enzyme in PPP, was also reduced in the hippocampus of STZ-treated mice (Figure 1F). *In vitro* experiments also showed that the expression of TIGAR and G6PD were reduced in both high glucose-treated primary neuron and Neuro-2a (Figures 1G,H). Our data indicated that the PPP pathway might be inhibited in hyperglycemia mice.

### Overexpression of TIGAR Alleviated High Glucose-Induced Neuronal Apoptosis in Primary Neurons

**Figure 2:**
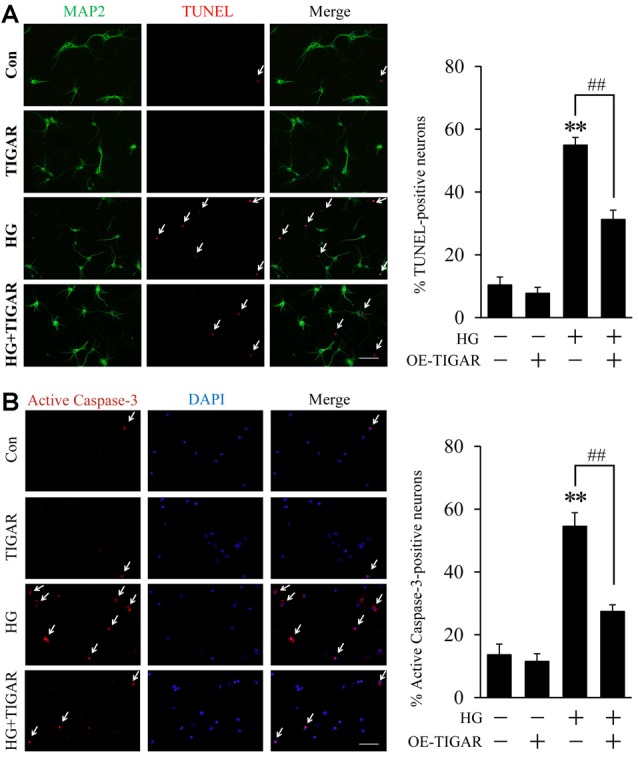
TIGAR reduced high glucose-induced neuronal apoptosis. **(A)** TUNEL and MAP2 staining and quantification of TUNEL-positive primary neurons in each group (*n* = 9 per group). **(B)** Immunofluorescent staining of active Caspase-3 and quantification of active Caspase-3-positive cells in each group (*n* = 9 per group). Scale bar = 50 μm. **p < 0.01 vs. the control group, ^##^*p* < 0.01 vs. the high glucose (HG) group, two-way ANOVA.

To investigate the effect of TIGAR in high glucose-induced neuronal apoptosis, overexpression of TIGAR by lentivirus (Lenti-TIGAR) was performed in primary neurons. The infection efficiency of Lenti-TIGAR was first investigated in primary neurons (Supplementary Figures 1B,D). TUNEL staining showed that high glucose increased the number of TUNEL-positive cells. The expression of MAP2 (neuronal marker) was significantly decreased in apoptotic cells. Compared to the high glucose (HG) group, overexpression of TIGAR significantly reduced the number of TUNEL-positive cells induced by high glucose (Figure 2A). We also observed the activation of Caspase-3 in high glucose-treated neurons. In Figure 2B, the number of active Caspase-3-positive cells was obviously attenuated after overexpression of TIGAR. The above data suggested that TIGAR might protect neurons from high glucose-induced apoptosis.

### TIGAR Rescued High Glucose-Induced Autophagy Impairment

**Figure 3:**
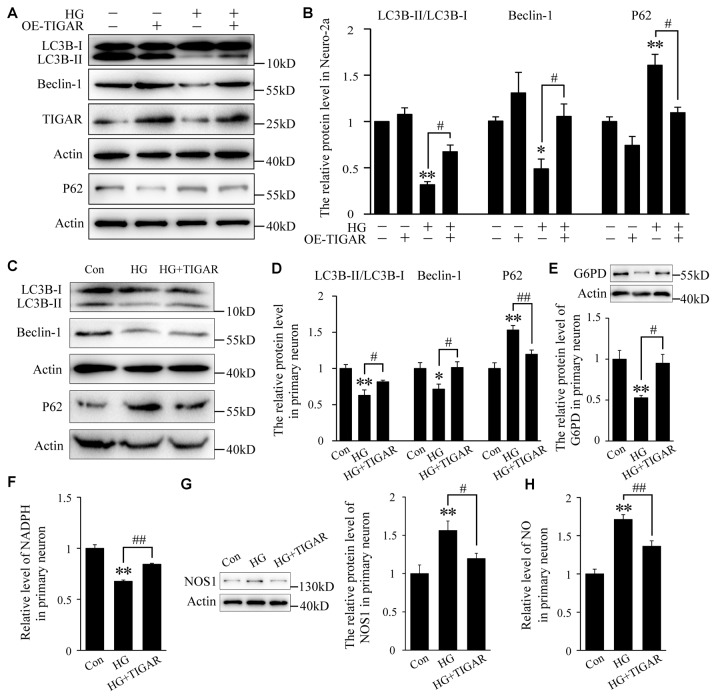
TIGAR ameliorated high glucose-mediated autophagy impairment. **(A,B)** Western blot analysis of LC3B, Beclin-1, TIGAR and P62 after treatment with Lenti-TIGAR and 50 mM glucose in Neuro-2a cells (*n* = 4 per group). **(C,D)** Western blot analysis of LC3B, Beclin-1 and P62 in primary neuron in each group (*n* = 4 per group). **(E)** Western blot analysis of G6PD in each group (*n* = 4 per group). **(F)** The relative level of NADPH in primary neuron (*n* = 6 per group). **(G)** Western blot analysis of nitric oxide synthase 1 (NOS1) in each group (*n* = 4 per group). **(H)** The relative level of NO in primary neurons (*n* = 6 per group). *p < 0.05, **p < 0.01 vs. the control group, ^#^*p* < 0.05, ^##^*p* < 0.01 vs. the HG group, one-way or two-way ANOVA.

Autophagy impairment has been found in the hippocampus of diabetic mice. To investigate the role of TIGAR in high glucose-induced autophagy dysfunction, the protein levels of LC3B, Beclin-1 and P62 were analyzed. The transfection efficiency of TIGAR plasmid was first investigated in Neuro-2a cells (Supplementary Figure 1C). In Figure 3, the ratio of LC3B-II/LC3B-I and the protein level of Beclin-1 were decreased after stimulation of high glucose in Neuro-2a cells. Lenti-TIGAR significantly rescued high glucose-induced decreases in the ratio of LC3B-II/LC3B-I and Beclin-1 protein (Figures 3A,B). In addition, the expression of P62, a cargo receptor for autophagic degradation, is accumulated during high glucose conditions, which was remarkably reversed by overexpression of TIGAR. The above results suggest that overexpression of TIGAR rescued autophagic impairments exposed to high glucose. In cultured primary neurons, overexpression of TIGAR also up-regulated autophagy flux under high glucose conditions (Figures 3C,D). In addition, overexpression of TIGAR increased the expression of G6PD and the level of NADPH (Figures 3E,F) in high glucose-treated primary neurons, suggesting that TIGAR might enhance the PPP pathway in neurons. Previous studies revealed that NOS1, a main enzyme to produce NO, plays a pivotal role in high glucose-induced autophagic flux impairment (Li et al., 2017). In our experiments, the expression of NOS1 was up-regulated in high glucose-treated primary neurons and significantly decreased after treatment of Lenti-TIGAR (Figure 3G). The increased generation of intracellular NO exposed to high glucose was largely prevented by Lenti-TIGAR (Figure 3H).

### TIGAR Protected High Glucose-Induced Apoptosis *via* Autophagy in Neuro-2a Cells

**Figure 4:**
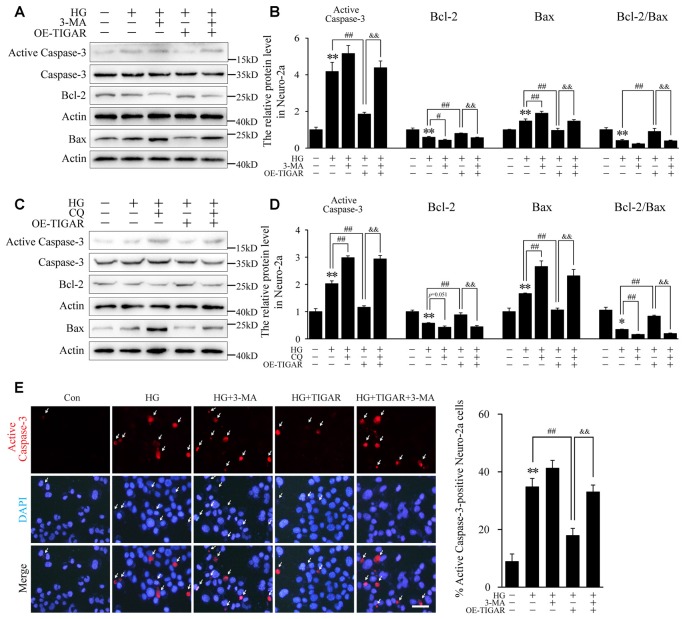
TIGAR reduced high glucose-induced apoptosis through regulating autophagy in Neuro-2a cells. **(A–D)** Autophagy inhibitors 3-MA and chloroquine diphosphate (CQ) abolished the effect of TIGAR on neuronal apoptosis in high glucose conditions. Western blot analysis of Bcl-2, Bax, active caspase-3 and total Caspase-3 under high glucose condition in each group (*n* = 5 per group). **(E)** Immunofluorescent staining of active Caspase-3 and quantification of active Caspase-3-positive cells in each group (*n* = 9 per group). Scale bar = 40 μm. *p < 0.05, **p < 0.01 vs. the control group, ^#^*p* <0.05, ^##^*p* < 0.01 vs. the HG group, ^&&^*p* < 0.01 vs. the HG+TIGAR group, two-way ANOVA.

3-MA and chloroquine, inhibitors of autophagy, were used to investigate the protective mechanism of TIGAR on neuronal apoptosis. Western blotting showed that the decrease of Bax and the increase of Bcl-2 in the Lenti-TIGAR-treated group were blocked after stimulation of 3-MA or chloroquine under high glucose condition (Figures 4A–D). 3-MA and chloroquine also reversed Lenti-TIGAR-decreased activation of Caspase-3. In addition, TIGAR plasmid was transfected into Neuro-2a to investigate the activity of Caspase-3 by immunofluorescent staining. We found that overexpression of TIGAR decreased the number of active Caspase-3-positive cells. Importantly, treatment of 3-MA blocked the effect of TIGAR on the activation of Caspase-3 (Figure 4E). Our data demonstrated that TIGAR prevented high glucose-induced neuronal apoptosis *via* the regulation of autophagy.

### TIGAR Ameliorated STZ-Induced Autophagy Impairment and Memory Loss in Mice

**Figure 5:**
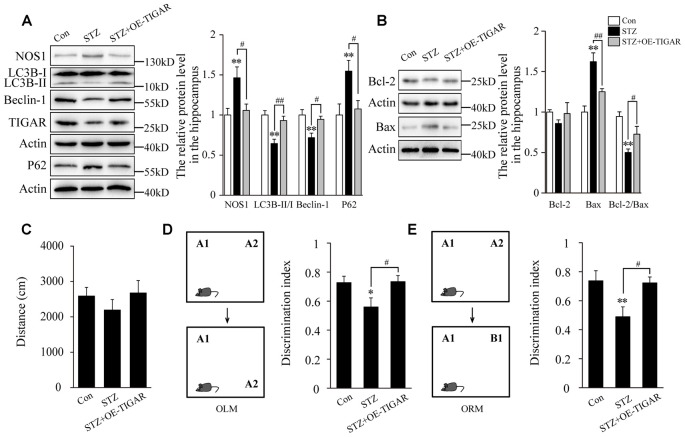
Overexpression of TIGAR reduced STZ-induced autophagy impairment and memory loss in mice. **(A)** Western blot analysis of NOS1, LC3B, Beclin-1, TIGAR and P62 after intraperitoneal injection of STZ and microinjection of Lenti-TIGAR in the hippocampus of mice (*n* = 5 per group). **(B)** Western blot analysis of Bcl-2 and Bax in the hippocampus of each group (*n* = 5 per group). **(C)** Locomotor activity was quantified 4 weeks after microinjection of Lenti-GFP or Lenti-TIGAR into the hippocampus of mice (*n* = 7 per group). **(D)** Schematic representation of the location-dependent object recognition memory (ORM) test (Left). Quantification of discrimination index in each group (*n* = 7 per group; Right). **(E)** Schematic representation of the novel ORM test (Left). Quantification of discrimination index in each group (*n* = 7 per group; Right). *p < 0.05, **p < 0.01 vs. the control group, ^#^*p* < 0.05, ^##^*p* < 0.01 vs. the STZ group, one-way ANOVA.

Evidence has proved that cognitive impairment in diabetes is closely related to hippocampal neuronal apoptosis (Shi et al., 2018). Therefore, we investigate the effect of TIGAR on STZ-induced neuronal apoptosis and memory loss in the hippocampus of mice. In Figure 5A, our data showed that injection of Lenti-TIGAR in the hippocampus significantly reduced STZ-induced increase of NOS1 protein and autophagy impairment. Compared with STZ-treated mice, the level of Bax was decreased and the ratio of Bcl-2/Bax was reversed in the hippocampus after overexpression of TIGAR (Figure 5B), suggesting that TIGAR reduced STZ-induced neuronal apoptosis in the hippocampus of mice. Furthermore, we investigated mouse memory-related behaviors in each group. STZ and microinjection of TIGAR showed no significant effect on mouse locomotor activity (Figure 5C). In ORM and OLM tests, STZ mice exhibited decreased discrimination ability compared with control mice, which was significantly rescued in Lenti-TIGAR-injected mice (Figures 5D,E). All the above results suggest that TIGAR might prevent high glucose-induced neuronal apoptosis and ameliorate cognitive impairment in diabetes.

## Discussion

Hyperglycemia-induced glucotoxicity is the leading cause of diabetic neuropathy (Vincent et al., 2004, 2011; Edwards et al., 2008). Full understanding and intervention of aberrant glucose metabolic pathways may become a potential therapeutic target for diabetic neuropathy. In the present study, we focus on the significance of TIGAR on neuronal glucose metabolism and provide several new insights into the effect of TIGAR on hyperglycemia-induced neuronal apoptosis. First, TIGAR was decreased in the hippocampus of diabetic mice and high glucose-treated neurons. Overexpression of TIGAR protected hyperglycemia-induced neuronal apoptosis. Second, TIGAR reduced the expression of NOS1 and ameliorated high glucose-induced autophagy impairments. Third, overexpression of TIGAR in the hippocampus ameliorated STZ-induced autophagy impairment and memory loss in mice. Our work revealed a novel mechanism linking the glycolysis regulator TIGAR with neuronal apoptosis in hyperglycemia conditions and indicated that TIGAR might prevent neuronal apoptosis *via* regulation of the autophagy process.

Diabetes and its complications are typical metabolic diseases originated from abnormal glucose metabolism. In the central nervous system, PPP and mitochondrial tricarboxylic acid cycles (TCA) and oxidative phosphorylation are two primary glucose consumption pathways in neurons. The PPP pathway plays pivotal roles in maintaining the bioenergetic and antioxidant status of neurons, while the TCA and oxidative phosphorylation pathways act as energy factories of ATP synthesis to satisfy neuronal physiological activity. Under hyperglycemic conditions, cells may shuttle most of the excess glucose into the mitochondria and produce an excess of reactive oxygen species (Leinninger et al., 2006). Studies have proved mitochondrial oxidative stress as one of the main causes leading to neuronal damage (Leinninger et al., 2006; Vincent et al., 2011; Yerra and Kumar, 2017). Conversely, the expression and enzymatic activity of G6PD are decreased in the dorsal root ganglions of STZ-induced diabetic mice and high glucose-treated PC-12 cells (Okouchi et al., 2005; Sun et al., 2019). In the present study, both TIGAR and G6PD were decreased in the hippocampus of STZ-treated mice and high glucose-stimulated neurons. This suggested that chronic hyperglycemia might increase mitochondrial oxidative stress and simultaneously inhibit the antioxidant PPP pathway. The aberrant metabolic pathway may further accelerate the process of neuron injury.

TIGAR has been proved as an important regulator of glycolysis and PPP (Li et al., 2014). Emerging evidence has proved that TIGAR plays neuroprotective effects in many neurological diseases (Zhou et al., 2016; Liu et al., 2018). Cisplatin, an effective antineoplastic agent and widely used in multiple tumor patients, was able to damage spiral ganglion neurons and caused hearing loss. In the mouse cochlea, TIGAR can be activated by Wnt signaling and protect against cisplatin-induced spiral ganglion neuron damage *via* restricting intracellular ROS and Caspase-3 expression (Liu et al., 2018). In ischemic/reperfusion-induced neuronal damage, the antioxidant and anti-inflammatory effect of TIGAR has been proved to be largely dependent on its metabolic regulation (Chen et al., 2018). In our study, TIGAR obviously decreased high glucose-induced apoptosis in both primary neurons and Neuro-2a cells. We found that overexpression of TIGAR reversed high glucose-induced decrease of G6PD and NADPH levels, suggesting that the anti-apoptotic function of TIGAR may rely on the regulation of the PPP pathway.

Mechanically, our data showed that TIGAR rescued high glucose-impaired autophagy activity. The function of autophagy in neurological diseases is sophisticated and remains controversial. Some investigators proved that autophagy activation caused self-attacking and cell death (Uchiyama et al., 2008; Qin et al., 2010; Zhang et al., 2019). Conversely, others believed that autophagy is a physiology process and inhibition of autophagy may induce neuronal apoptosis (Zhang et al., 2013; Caccamo et al., 2017; Li et al., 2017). This suggested that a moderate effect of autophagy on self-cleaning may be necessary for neuronal activity while an overwhelming increase of autophagy will be destructive and may lead to neuronal injury. Nevertheless, autophagy impairments have been observed in the hippocampus of diabetic mice and high glucose-treated neurons (Xue et al., 2016; Yerra and Kumar, 2017). Up-regulation of autophagy flux attenuated chronic hyperglycemia-induced neuronal loss (Li et al., 2017). In this study, TIGAR significantly increased the autophagic flux in both high glucose-treated primary neurons and Neuro-2a cells. Furthermore, 3-MA and chloroquine, inhibitors of autophagy, blocked the protective effect of TIGAR on high glucose-induced apoptosis in Neuro-2a cells. Therefore, we demonstrated that TIGAR might attenuate high glucose-induced neuronal apoptosis *via* regulation of autophagy.

NOS1/nNOS, an enzyme to produce NO, plays an important role in high glucose-induced autophagic flux impairment (Li et al., 2017). The increased production of NO enhanced S-nitrosation of ATG4 and impaired autophagosome biogenesis, and finally leads to neurotoxicity. An inverse association between the activity of G6PD and muscle-specific nNOS was observed in skeletal muscle cells (Lee-Young et al., 2016). Moreover, overexpression of G6PD displayed remarkable resistance against NO-mediated apoptosis in PC12 cells (García-Nogales et al., 2003). However, the effect of TIGAR on the expression of NOS1 is unknown. Our data showed that TIGAR prevented high glucose-induced expression of NOS1 and NO generation in primary neurons, suggesting that the protective effect of TIGAR on autophagy may be associated with the regulation of NOS1.

Under nutrient starvation or metabolic stress conditions, TIGAR acts as a negative regulator of autophagy and plays anti-apoptotic roles in cancer cells (Bensaad et al., 2009; Xie et al., 2014). In ischemic stroke, oxygen and glucose deprivation in the brain might induce large neuronal injury and excessive activation of autophagy. TIGAR inhibited autophagy by increasing phosphorylation of mTOR and S6KP70 and reduced neuronal damage (Zhang et al., 2019). However, under hyperglycemic conditions, TIGAR displayed an opposite effect. TIGAR could protect against high glucose-induced autophagy impairment. This suggested that TIGAR may have a dual role in regulating autophagy under different metabolic conditions. As reported, TIGAR is a key glycolysis-related enzyme and dominates many signaling pathways. Under different stimulation, TIGAR may influence a different signaling pathway. In addition, the role of autophagy in neuron injury is still controversial. TIGAR might maintain autophagic homeostasis through rebuilding impaired autophagy and suppressing excessive autophagy by multiple pathways.

Finally, our present work demonstrated that TIGAR alleviated STZ-induced autophagy impairment, neuronal apoptosis and memory loss in the hippocampus. The protective effect of TIGAR on neuronal survival may be largely dependent on its regulation of autophagy. Future clinical studies may identify TIGAR as a therapeutic target for the treatment of diabetic neuropathy and cognitive impairment.

## Data Availability

All datasets generated for this study are included in the manuscript and/or the Supplementary Files.

## Ethics Statement

All procedures conformed to the National Institutes of Health Guide for the Care and Use of Laboratory Animals and were allowed by the Institutional Animal Care and Use Committees of Shandong University.

## Author Contributions

This work was mainly completed by WZ. YY, JL and DW participated in the establishment of STZ-induced diabetic model and *in vivo* experiments. MZ, ZY and AP contributed to the neuronal cell culture and *in vitro* experiments. LK was involved in the conception and design, manuscript writing, financial support and final approval of the manuscript.

## Conflict of Interest Statement

The authors declare that the research was conducted in the absence of any commercial or financial relationships that could be construed as a potential conflict of interest.
