# Supplementary material for: TIGAR Attenuates High Glucose-Induced Neuronal Apoptosis via an Autophagy Pathway
Source: Front Mol Neurosci. 2019 Aug 13;12:193. doi: 10.3389/fnmol.2019.00193 (PMC6700368; doi:10.3389/fnmol.2019.00193)
Supplement: Supplementary file 1 [file Data_Sheet_1.docx]

**Supplementary material**

**TIGAR attenuates high glucose-induced neuronal apoptosis *via* an autophagy pathway**


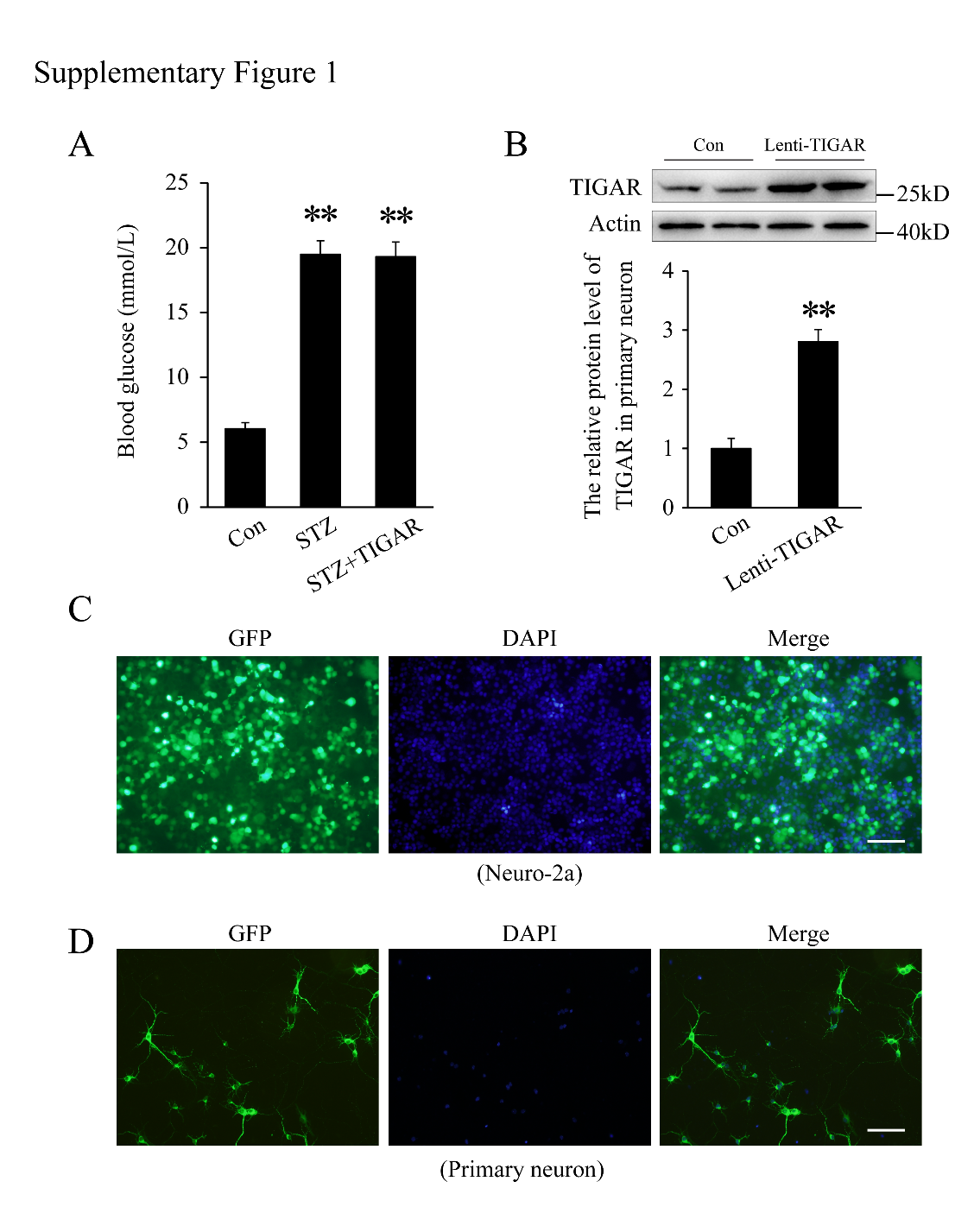


Supplementary Figure. A, The blood glucose concentration of mice in control, STZ and STZ+Lenti-TIGAR groups (n = 7 per group). B, The overexpression efficiency of Lenti-TIGAR in primary neuron (n = 4 per group), ***p* < 0.01. C, The transfection efficiency of pUltra-TIGAR in Neuro-2a was up to 90%. scale bar = 200 μm. D, The infection efficiency of Lenti-TIGAR in primary neuron was nearly 75%. scale bar = 100 μm.
